# Accuracy of toric intraocular lens implantation using automated vs manual marking

**DOI:** 10.1186/s12886-019-1175-1

**Published:** 2019-08-03

**Authors:** Eun Chul Kim, Kyu Yeon Hwang, Sung A. Lim, Rowoon Yi, Choun-Ki Joo

**Affiliations:** Department of Ophthalmology and Visual Science, College of Medicine, Catholic University of Korea, Seoul St. Mary’s Hospital, #222 Banpo-daero, Seocho-gu, Seoul, 137-701 South Korea

**Keywords:** Astigmatism, SensoMotoric instruments (SMI), Toric intraocular lens (TIOL), Steep axis

## Abstract

**Background:**

Accurate alignment of toric intraocular lens (TIOL) to steep corneal astigmatic axis is important to achieve effective postoperative results. The authors compare the accuracy of astigmatism correction using automated and manual marking in TIOL implantation during cataract surgery.

**Methods:**

One hundred thirty-two eyes with nuclear density from Grade 2 to 4 were randomly subdivided into 2 groups (automated and manual marking). All patients underwent manual marking and the steep axis was compared to SensoMotoric Instruments (SMI). After phacoemulsification, 62 patients underwent toric IOL implantation using the SMI and 70 patients underwent toric IOL implantation using manual marking. Intraoperative measurement was the steep axis difference. Clinical measurements included preoperative and postoperative best corrected visual acuity (BCVA), and TIOL axis.

**Results:**

The intraoperative steep axis difference between SMI and manual marking was 7.86 ± 6.4 degrees. The difference between the preoperative steep axis and the postoperative TIOL axis using SMI (3.63 ± 1.12 degrees) was significantly lower than that using manual marking (8.29 ± 2.23 degrees) (*P* < 0.05).

**Conclusions:**

The steep axis measurements may be different when using SMI vs. manual marking. The SMI is more accurate than manual marking for TIOL implantation during cataract surgery.

**Trial registration:**

Current Controlled Trials ISRCTN12294725, Retrospectively registered, on 20 July 2018.

## Background

With developments in cataract surgery, only correction of refractive errors with removal of the opaque lens and implantation of an intraocular lens is not the major goal of the cataract surgery. An estimated 35% of cataract patients have 1.00 diopter (D) or more than 1.00 diopter of preexisting corneal astigmatism [1], and 15 to 20% of them have 1.5 D or more than 1.5 D of corneal astigmatism [2].

Limbal corneal relaxing incisions and implantation of a toric intraocular lens (IOL) have been used for correction of astigmatism in cataract patients [3, 4].

Because 1° of off-axis rotation results in a loss of up to 3.3% of lens cylinder power [5], marking the accurate axis is most important for successful toric IOL implantation. Several marking methods have been used, for example, marking at the 3-, 6-, and 9-o’clock positions using a toric reference marker and at the 3 and 9 o’clock positions using a horizontal slit beam in the slit lamp [6]. An anterior segment photograph was used to identify several reference vessel points and axis marking points [7]. Digital overlay imaging was also used to evaluate the alignment of toric IOL [8].

Recently, an eye tracking systems called SensoMotoric Instruments (SMI, Teltow, Germany) has been used to visualize the steep corneal axis through the operating microscope during toric IOL implantation.

To the best of our knowledge, there are no studies evaluating the efficacy of SMI in cataract surgery with toric IOL implantation.

Here in, we compare the outcomes of toric IOL implantation marked with SMI and manual marking 2technique in the cataract patients with corneal astigmatism.

## Methods

This prospective randomized study comprised 132 eyes of 132 patients with cataract and coexisting corneal astigmatism more than 1.5 diopters (D) who were randomly assigned to undergo phacoemulsification and posterior chamber toric intraocular lens implantation at the Seoul St. Mary’s Hospital between February 2014 and December 2017. The study protocol followed the guidelines of the Declaration of Helsinki and the institutional review board of Bucheon St. Mary’s Hospital. Patients provided written informed consent after receiving an explanation of the surgical systems used in the study.

One hundred thirty-two eyes were randomly divided into two groups (SMI and manual marking). All patients underwent manual marking and the steep axis was compared to SMI intraoperatively. After phacoemulsification, 62 patients underwent toric IOL implantation using SMI (group 1) and 70 patients underwent toric IOL implantation using manual marking (group 2).

Corneal pathology, pseudoexfoliation, history of ocular trauma and intraoperative complications such as posterior lens capsule rupture, lens dislocation, and ocular inflammation were excluded.

Uncorrected visual acuity (UCVA), best corrected visual acuity (BCVA), keratometer, refractometer, and corneal topography by a Scheimpflug imaging system (Pentacam; Oculus, Wetzlar, Germany) were assessed preoperatively.

### Intraoperative & postoperative measurements

Intraoperative measurements included the steep axis difference between SMI and manual marking. The postoperative parameters measured at 1-day postoperatively, 1-month postoperatively, and 2-months postoperatively were uncorrected visual acuity (UCVA), best corrected visual acuity (BCVA), keratometer, refractometer, and corneal topography by a Scheimpflug imaging system (Pentacam). The visual acuity measurements had been recorded by masked personnel.

### Surgical technique

For SMI marking, reference image of the steep axis was taken using a reference unit in the outpatient department. The reference image and data of the steep axis were transferred to the SMI system in the operating room.

For manual marking, the axis-marking procedure was performed with topical anesthesia. The patient was seated at the surgical table and instructed to gaze at a distant target. Using a toric reference marker (AE-2793S; ASICO LLC, Westmont, Illinois), the corneal limbus was marked at the 3-, 6-, and 9-o’clock positions. Next, with the patient lying on the surgical table, the steep axis was marked using a Mendez ring (K3–800; Katena, Denville, New Jersey). The steep axis difference between SMI and manual marking was measured in all 132 eyes.

Phacoemulsification was performed by the same surgeon (C.K.J.) using the Intrepid Infiniti system as described previous publication [9]. After phacoemulsification, sodium hyaluronate 1% (Healon®) was injected into the anterior chamber, and a toric intraocular lens (IOL) was inserted in the capsular bag. A SN6AN (Alcon, Ft. Worth, TX, USA) was implanted in the capsular bag using an injector system. In 62 randomized patients, the axis marks on the toric IOLs were aligned with the steep corneal meridian determined by the SMI, and in another 70 randomized patients, it was determined by manual marking under the protection of an ophthalmic viscosurgical device (OVD), which was subsequently removed through aspiration. The wound was not sutured.

### Statistical analysis

All data are expressed as the mean ± standard deviation. Pairwise comparisons of treatment group categorical variables were performed using the Mann-Whitney U test and continuous variables were analyzed using the unpaired t test. The analyses were performed using SPSS for Windows software (version 16.0, SPSS, Inc.). A *P* value of less than 0.05 was considered statistically significant.

## Results

### Preoperative parameters

Table 1 shows the characteristics of the patients in each group. There were no statistically significant differences between the two groups according to age, preoperative mean astigmatism, and uncorrected and best corrected visual acuity (UCVA & BCVA) (*P* > 0.05).

**Table 1:** Preoperative clinical data

	Group 1 (SMI)	Group 2 (Manual technique)
Total patients (Eyes)	62 (62)	70 (70)
Age	58.22 ± 12.84	61.63 ± 16.15
Mean Asigmatism (Diopter)	2.68 ± 1.05	2.46 ± 0.97
Uncorrected visual acuity (logMAR)	0.65 ± 0.24	0.63 ± 0.21
Best corrected visual acuity (logMAR)	0.32 ± 0.12	0.34 ± 0.11
*P* value	> 0.05	> 0.05

Data represent mean ± standard deviation

SMI: SensoMotoric Instruments

There was no statistically significant difference in initial characteristics between the two groups by the unpaired *t* test (*P* > 0.05, unpaired *t*-test)

### Intraoperative parameters

**Fig. 1:**
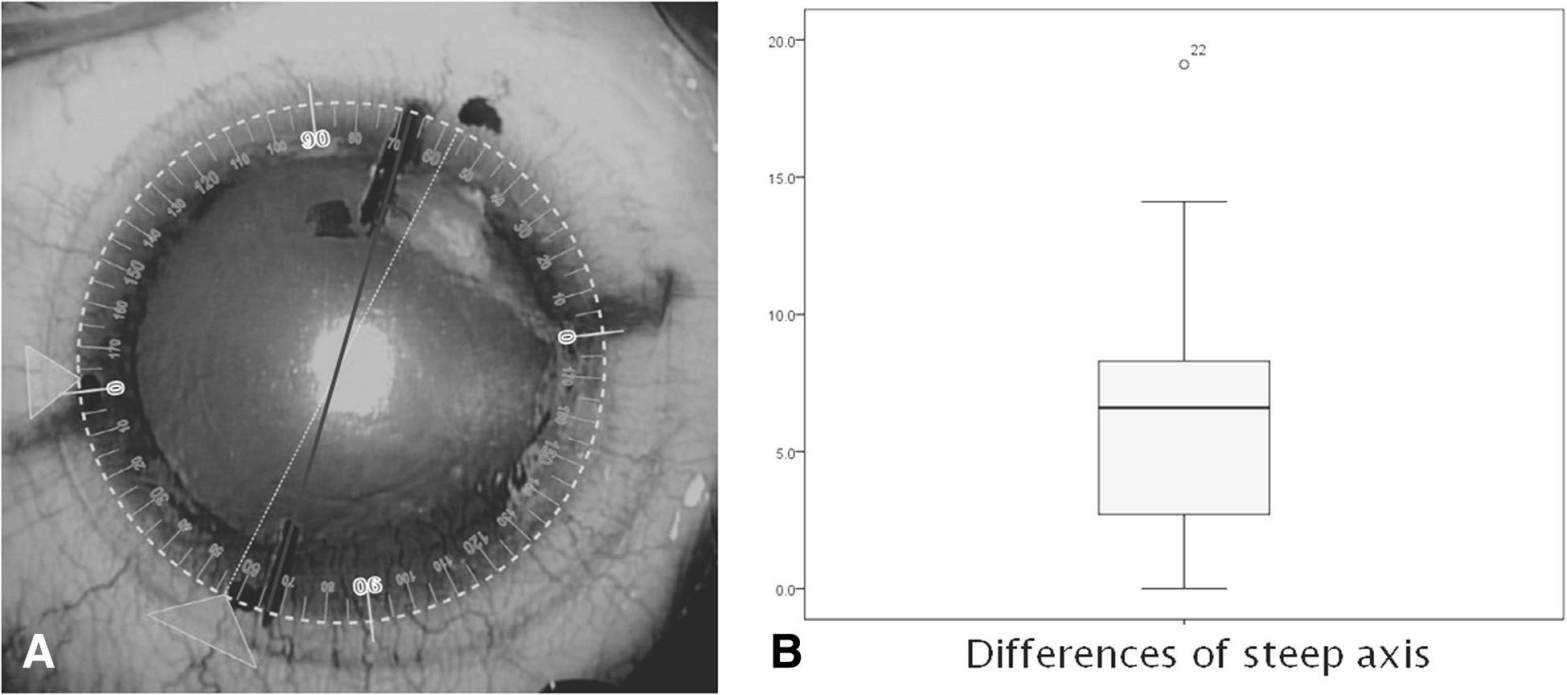
Intraoperative astigmatic axis difference between SensoMotoric Instruments and manual marking. **a**: dot line-SMI axis, thick line-manual axis. **b**: The difference in the steep astigmatic axis is 7.86 ± 6.4 degrees. There was a significant axis difference between the two groups (*P* < 0.05 by the Mann-Whitney U test)

The intraoperative steep axis difference between SMI and manual marking was 7.86 ± 6.4 degrees. There was a significant axis difference between the two groups (*P* < 0.05) (Fig. 1).

### Postoperative TIOL axis differences

**Fig. 2:**
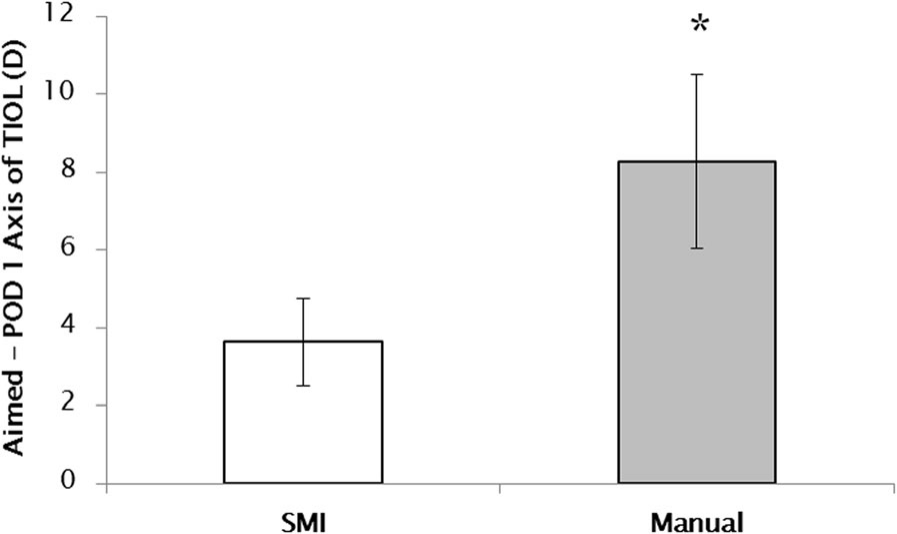
Axis difference in Toric Intraocular Lens (TIOL) between the aimed and postoperative axis 1 day after cataract surgery. The difference between the preoperative steep axis and the 1 day postoperative TIOL axis using SMI was significantly lower than that using manual marking (P < 0.05 by the Mann-Whitney U test)

**Fig. 3:**
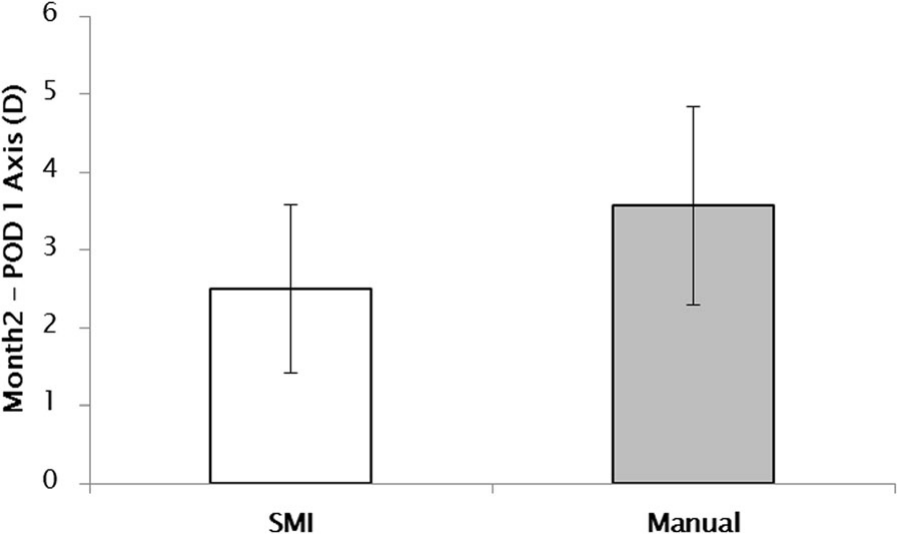
Axis rotation of the Toric Intraocular Lens (TIOL) between postoperative 1 day and 2 months after cataract surgery**.** There was no significant difference in TIOL rotation between SensoMotoric Instruments and manual marking (*P* > 0.05 by the Mann-Whitney U test)

The difference between the preoperative steep axis and the 1-day postoperative TIOL axis using SMI (3.63 ± 1.12 degrees) was significantly lower than that using manual marking (8.29 ± 2.23 degrees) (*P* < 0.05) (Fig. 2). The axis difference of group 1 (4.75 ± 1.37 degrees) was significantly lower than that of group 2 at the postoperative 2 months (8.93 ± 2.17 degrees) (*: *P* < 0.05) (Table 2). There was no significant difference in TIOL rotation between the two groups (*P* > 0.05) (Fig. 3).

**Table 2:** Postoperative results after 2 months

	Group 1 (SMI)	Group 2 (Manual technique)
Total patients (Eyes)	62 (62)	70 (70)
Mean Asigmatism (Diopter)	0.52 ± 0.32	0.59 ± 0.30
*Axis difference† (Degrees)	4.75 ± 1.37	8.93 ± 2.17
*Uncorrected visual acuity (logMAR)	0.07 ± 0.02	0.10 ± 0.03
Best corrected visual acuity (logMAR)	0.02 ± 0.01	0.02 ± 0.01

Data represent mean ± standard deviation

*SMI* SensoMotoric Instruments

Axis difference†: The difference between the preoperative steep axis and the postoperative TIOL axis

The axis difference of group 1 was significantly lower than that of group 2 (*: *P* < 0.05). And, the uncorrected visual acuity (logMAR) of group 1 was significantly higher than that of group 2 (*: *P* < 0.05)

### Uncorrected and best corrected visual acuity (log MAR)

Uncorrected visual acuity (logMAR) in the SMI group was significantly higher than that in the manual marking group (*P* < 0.05). But, there was no significant difference in the BCVA between the two groups (*P* > 0.05) (Table 2).

## Discussion

Microincision and small incision cataract surgery enables the almost astigmatically neutral phacoemulsification incisions [10]. Newly developed intraocular designs and materials have improved the stability and predictability of Intraocular Lenses (IOLs) [11].

Astigmatic error has become the most important cause of low uncorrected visual acuity, as the phacoemulsification technique has improved. Astigmatism less than 0.5 D did not degrade visual acuity after cataract surgery [12]. Patients with > 0.75 D of corneal astigmatism had better visual outcome with implantation of toric IOLs compared to monofocal IOLs [13].

Because of the innovations in IOL technology and phacoemulsification technique, implantation of toric IOLs is the best method for correction of large corneal astigmatism more than 1.5 Diopters in patients requiring cataract surgery [11].

Accurate alignment of toric IOL to steep corneal astigmatic axis is important to achieve effective postoperative results [14]. Inaccurate alignment of the toric IOL occurs due to wrong alignment during the operation or postoperative IOL rotation [14].

Many methods are used to mark the steep corneal astigmatic axis. When the patient is sitting, the preoperative marking of the horizontal corneal meridian is the most important step [14]. Marking of the horizontal meridian can be done by slit lamp-assisted marking with a horizontal slit beam, a pendular marker, and a nonpendular marker [6, 15].

Nowadays, digital image guidance such as SMI ensures accurate toric IOL alignment without manual marking during cataract surgery.

Elhofi AH et al. compared the clinical outcome of digital and manual marking for toric intraocular lens (IOL) alignment [14]. The mean postoperative uncorrected distance visual acuity (UCDVA) for the digital-marking group (0.12 + 0.12 logMAR) was higher than that for the manual-marking group (0.18 + 0.14 logMAR) (*P* = 0.104) [14]. The mean deviation from targeted induced astigmatism (TIA) for the digital-marking group (0.10 + 0.08 D) was lower than that for the manual-marking group (0.20 + 0.14 D) (*P* = 0.001) [14]. Also, the mean postoperative toric IOL misalignment measured by the slitlamp for the digital-marking group (2.48 + 1.968) was lower than that for the manual-marking group (4.338 + 2.728) (*P* = 0.003) [14].

However, there is no study comparing digital-marking and manual-marking in the same patients.

In our study, all patients underwent SMI and manual marking, and we compared manual marking to SMI intraoperatively. We calculated the mean axis difference between the two methods. There was a significant axis difference (7.86 ± 6.4 degrees) between the two groups (*P* < 0.05) (Fig. 1).

The accuracy of marking steep axis using SMI was significantly better than that using manual marking after 1 day (Fig. 2) and 2 months (Table 2) (*P* < 0.05). The visual acuity (logMAR) in the SMI group was also significantly better than that in the manual marking group at the postoperative 2 months (*P* < 0.05).

Atchison DA et al. described that small levels of crossed cylinder blur produces losses in visual acuity that are dependent on the cylinder axis [16]. The difference of the uncorrected visual acuity (logMAR) between two groups at the postoperative 2 months may occur because of the axis difference between the preoperative steep axis and the postoperative TIOL axis (Table 2).

To the best of our knowledge, this is the first study comparing manual marking and SMI in the same patients during the operation. Our results showed that SMI causes a large difference in the steep astigmatic axis compared to manual marking. Also, the postoperative effectiveness of the toric IOL axis and uncorrected visual acuity for SMI were better than those for manual marking. However, there was no significant difference in TIOL rotation and best corrected visual acuity between the two groups (*P* > 0.05).

The only limitation of this study was that we provided the results obtained by a single surgeon. A multivariate evaluation should be performed in the future.

## Conclusions

Accurate marking of the steep astigmatic axis is important to achieve accurate alignment of the toric IOL and good postoperative uncorrected visual acuity. SMI would be a better instrument that provides accurate preoperative marking, intraoperative toric IOL alignment, and better postoperative results.

## Data Availability

The datasets used and/or analyzed during the current study available from the corresponding author on reasonable request.

## Abbreviations

BCVA: Best corrected visual acuity
LogMAR: Logarithm of the Minimum Angle of Resolution
SMI: SensoMotoric Instruments
TIA: Targeted induced astigmatism
TIOL: Toric intraocular lens
UCDVA: Uncorrected distance visual acuity
UCVA: Uncorrected visual acuity
